# Characterization of a bent Laue double-crystal beam-expanding monochromator

**DOI:** 10.1107/S1600577517014059

**Published:** 2017-10-19

**Authors:** Mercedes Martinson, Nazanin Samadi, Xianbo Shi, Zunping Liu, Lahsen Assoufid, Dean Chapman

**Affiliations:** a Physics and Engineering Physics, University of Saskatchewan, 116 Science Place, Rm 163, Saskatoon, Saskatchewan, Canada S7N 5E2; b X-ray Science Division, Argonne National Laboratory, 9700 South Cass Avenue, Lemont, IL 60439, USA; c Canadian Light Source, 44 Innovation Boulevard, Saskatoon, Saskatchewan, Canada S7N 2V3

**Keywords:** beam expander, double bent Laue monochromator, Lang topography, Berg–Barrett topography, finite-element analysis

## Abstract

A bent Laue double-crystal monochromator system has been designed for vertically expanding the X-ray beam at the Canadian Light Source’s BioMedical Imaging and Therapy beamlines. Expansion by a factor of 12 has been achieved without deteriorating the transverse coherence of the beam, allowing phase-based imaging techniques to be performed with high flux and a large field of view. However, preliminary studies revealed a lack of uniformity in the beam, presumed to be caused by imperfect bending of the silicon crystal wafers used in the system. Results from finite-element analysis of the system predicted that the second crystal would be most severely affected and has been shown experimentally. It has been determined that the majority of the distortion occurs in the second crystal and is likely caused by an imperfection in the surface of the bending frame. Measurements were then taken to characterize the bending of the crystal using both mechanical and diffraction techniques. In particular, two techniques commonly used to map dislocations in crystal structures have been adapted to map local curvature of the bent crystals. One of these, a variation of Berg–Berrett topography, has been used to quantify the diffraction effects caused by the distortion of the crystal wafer. This technique produces a global mapping of the deviation of the diffraction angle relative to a perfect cylinder. This information is critical for improving bending and measuring tolerances of imperfections by correlating this mapping to areas of missing intensity in the beam.

## Abstract 2

A previously reported bent Laue double-crystal monochromator was found to have areas of missing intensity in the final X-ray beam. Measurements of the shape of the bent crystal wafers have been made using mechanical and diffraction methods to evaluate the crystal system and provide insight into potential methods of mitigating the non-uniformities in the beam.

## 1. Introduction   

**Figure 1:**
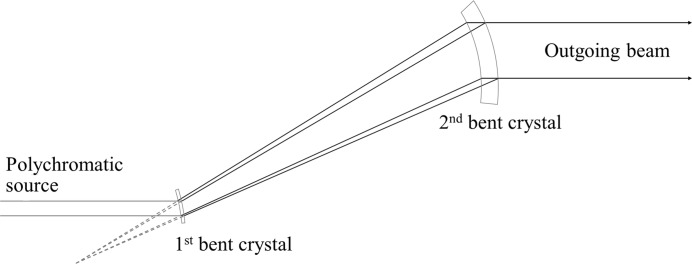
Schematic of bent Laue double-crystal system.

**Figure 2:**
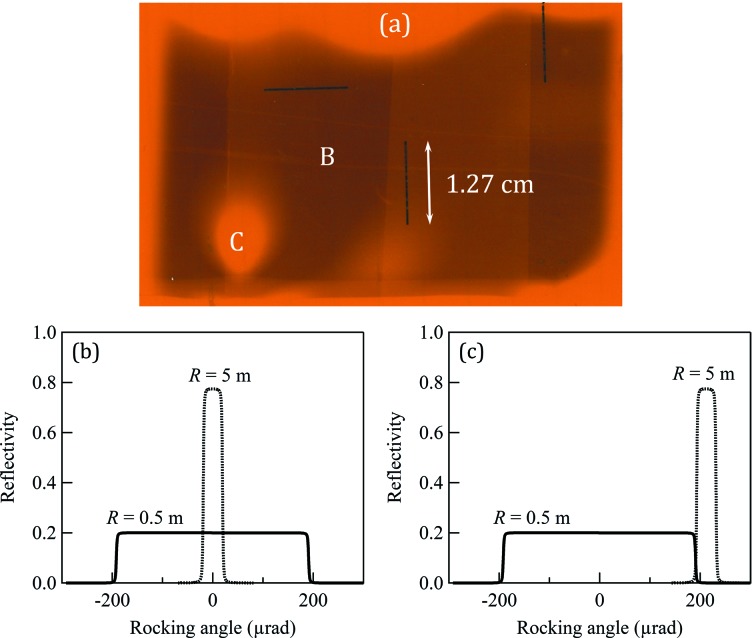
(*a*) Double-diffracted expanded beam showing intensity distribution due to distortion in the crystal. Areas B and C show low and high intensity, respectively. Corresponding reflectivity curves (*b*) overlapping and (*c*) failing to overlap.

The BioMedical Imaging and Therapy (BMIT) beamlines at the Canadian Light Source would greatly benefit from an increase in the vertical size of the X-ray beam, which would enable dynamic imaging of animal samples that are larger than currently possible with the 7 mm and 11 mm vertical heights of the bending-magnet and insertion-device beamlines, respectively. Preserving the quality of the transverse coherence while expanding the beam would enable phase imaging techniques in a field of view capable of completely covering many small animals, extending the beamline’s capabilities to functional dynamic imaging of soft tissue such as lungs. Making full use of the large animal imaging stage, a feature unique to this facility (Wysokinski *et al.*, 2007 ▸), similarly requires a larger field of view. Previous results (Martinson *et al.*, 2014 ▸, 2015 ▸) reported on the development of a phase-preserving bent Laue beam-expanding double-crystal monochromator: two silicon (Si) crystal wafers were cylindrically bent with the concave sides facing the X-ray beam and arranged with the geometrical foci of both crystals co-located and the diffraction planes parallel between each crystal as in Fig. 1 ▸. This system increased the vertical size of the X-ray beam by a factor of 12 without adversely affecting the transverse coherence in the diffraction plane. These initial experiments were made on the bending-magnet beamline (Wysokinski *et al.*, 2007 ▸). However, the intensity of the final beam was not uniform across the entire field of view (Fig. 2*a* ▸). In particular, one region was severely affected by a large ‘hole’ present in the beam. To overcome this problem, it was first necessary to characterize the bending of the bent crystal wafers. Measurements of the bent crystal mounted in the solid bending frame were taken using both mechanical and diffraction methods. The mechanical measurements indicated an area of the crystal with significant physical distortion that corresponds exactly to the location of the hole in the expanded beam. The diffraction measurements also clearly indicated a large area of distortion in the second crystal corresponding to both the hole in the beam and the area of physical distortion in the crystal surface. Finally, the effect of this distortion on the diffraction angles was carefully measured by analysing diffraction images produced by bent crystals in a variation of Berg–Barrett topography. This analysis technique represents a new way to characterize bent crystals used as optical components for many types of synchrotron beamlines. This characterization can then be used to help locate and reduce the distortions until they fall within a set tolerance.

## 2. Background   

**Figure 3:**
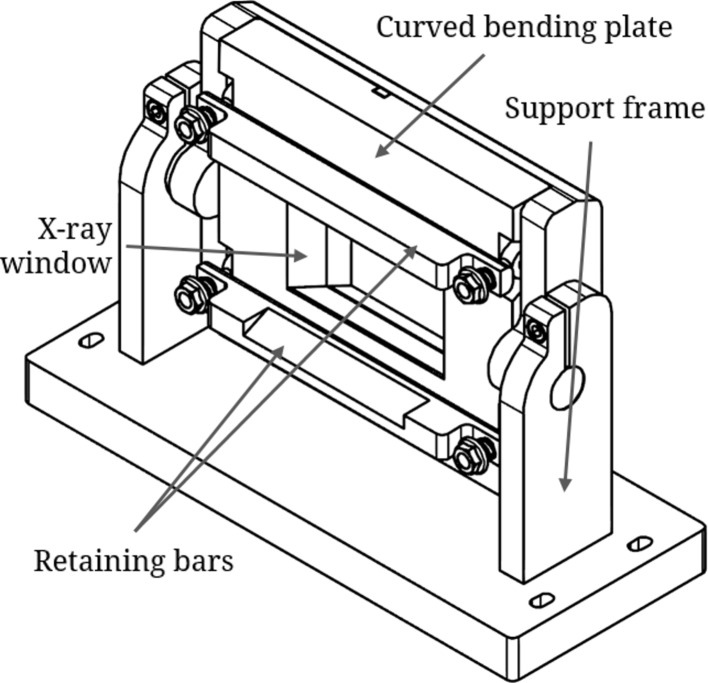
Crystal bending apparatus.

The crystals used in this work were Si(5,1,1) wafers with the (3,1,1)-type reflection aligned to the iodine *K*-edge (33.2 keV). Both crystals are 0.65 mm thick. The first crystal was bent to a radius of 0.5 m while the second crystal was bent to a radius of 5 m (see Table 1 ▸ for full parameters). The bending apparatus (Fig. 3 ▸) consists of a solid metal bending plate machined with a curvature of the appropriate bending radius (0.5 m and 5 m for the first and second crystals, respectively) such that the crystal will be cylindrically bent in the diffraction plane. The crystals are held in place and forced to the appropriate bending radius by solid steel retaining bars. The bending plates are mounted on support frames that can be attached to the rotating stages for crystal alignment, and that can also be rotated themselves within the supports for coarse alignment.

**Figure 4:**
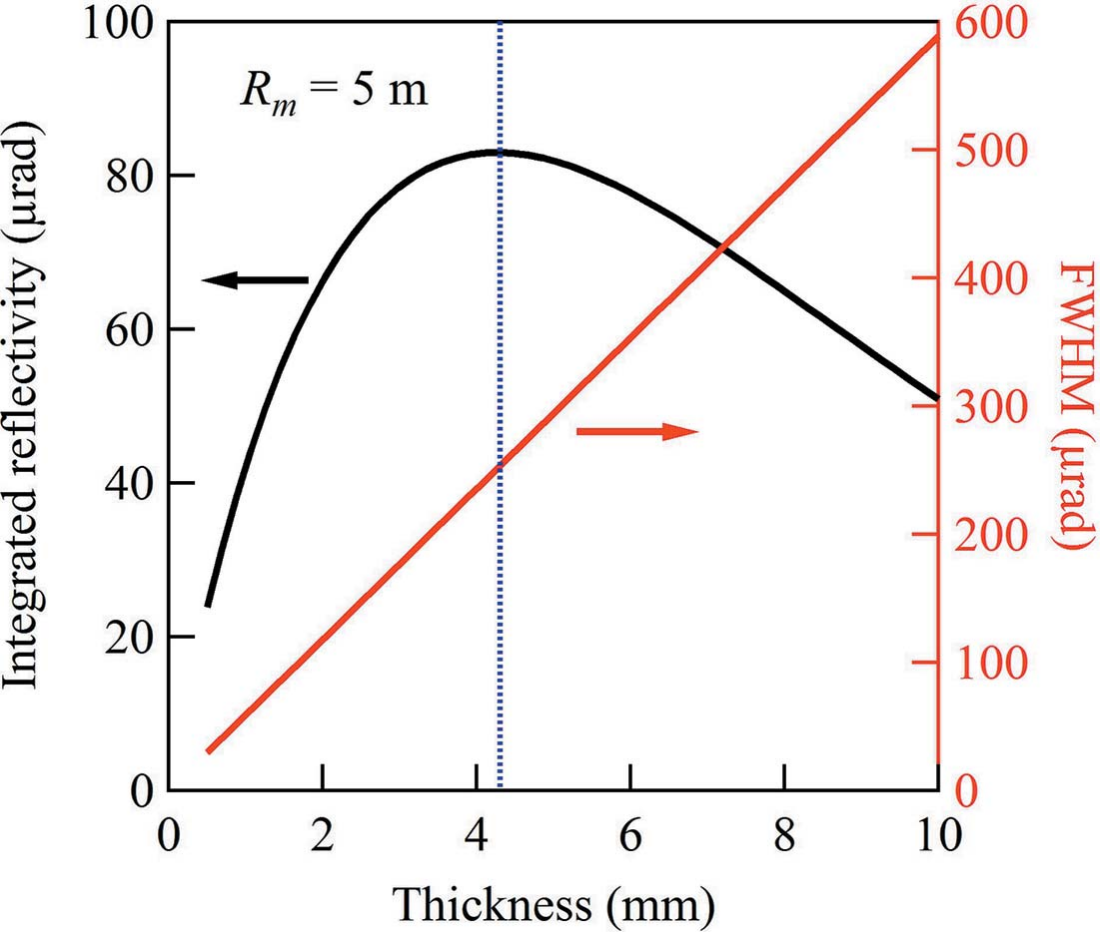
Integrated reflectivity and angular bandwidth at 33.2 keV of the expander crystals as a function of crystal thickness.

The reflectivity curves (or rocking curves) of the two crystals are calculated using the *Xcrystal* module (Sanchez del Rio *et al.*, 2015 ▸) in *XOP* (Sanchez del Rio & Dejus, 2011 ▸) and shown in Figs. 2(*b*) and 2(*c*) ▸. At 5 m bending radius, the width of the reflectivity curve (38 µrad FHWM) of the second crystal is about ten times narrower than that of the first crystal (380 µrad) at 0.5 m bending radius. The angular tolerance on the matching of the two crystals is determined by the reflectivity curve width of the first crystal. The area of missing intensity shown in Fig. 2(*a*) ▸ occurs because the reflectivity curve of the second crystal falls completely outside that of the first one (Fig. 2*c* ▸). To increase the efficiency of the system but keep the bandwidth of the first crystal, the second crystal needs to be much thicker. The maximum integrated reflectivity of the second crystal occurs at a thickness of 4.3 mm with a bandwidth of 250 µrad at 33.2 keV (Fig. 4 ▸). A crystal this thick could never be bent to the small radius required by the system, so the flexibility of the silicon wafers remains the most important factor in crystal thickness choice. As the degree of anticlastic bending also depends on the crystal thickness, this suggests a compromise between maximizing reflectivity in the diffraction plane and reducing anticlastic bending.

## 3. Finite-element analysis   

It is well known that anticlastic bending is an important factor whenever crystals are bent (Zontone & Comin, 1992 ▸). It was suggested that the lack of uniformity in the beam could be caused by a severe mismatch between crystals caused by anticlastic bending. This would lead to missing intensity along the side edges of the beam due to mismatch of the crystal planes; however, this effect would be mitigated as the anti­clastic bending should contribute a relatively small component to the diffraction angle. Finite-element analysis (FEA) was used to predict the expected shape of the crystals when bent with the solid frame bender. Using the design parameters for the bending frames (0.5 m and 5 m cylindrical slabs), the actual bend radii predicted for the crystals were 0.518 m and 5.06 m, respectively, with anticlastic bend radii of 51.4 m and 70.8 m. This indicates that the second crystal may be more adversely affected by anticlastic bending due to its larger bend radius and lower tolerance for small irregularities. As the anticlastic bend radii are on the same order of magnitude and perpendicular to the diffraction plane, we do not expect serious effects on intensity, despite the comparatively larger ratio between principle and anticlastic bend radii in the second crystal. Naturally, this analysis did not predict the hole of missing intensity as this was likely caused by imperfections in the physical bending frame.

## 4. Mechanical measurements   

**Figure 5:**
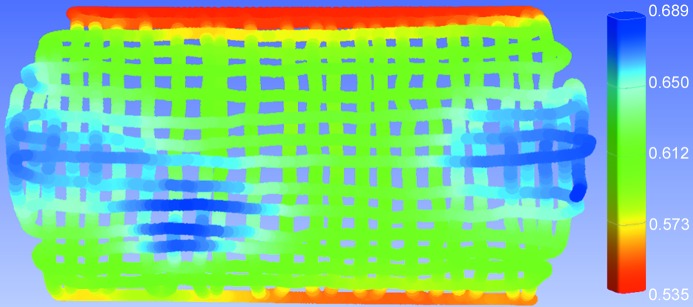
Three-dimensional physical mapping of 5 m bend radius crystal surface. The colour bar indicates the distance in mm from the bending surface to a flat plane fit slightly below the bending surface.

As we suspected imperfections in the physical bend, the natural first step was to measure the bent crystals mechanically using a FaroArm (FARO Technologies Ltd). Fitting these data to cylinders demonstrated that each crystal was within 1% of the desired bend radius. A colour mapping was created of the 5 m radius. The data were fit to a flat plane slightly below the bending surface and the distance from this plane to the bending surface was measured and mapped out in colour (Fig. 5 ▸). As the measurements were made by hand using a FaroArm, there are inconsistencies between runs, as indicated by the slight colour variations between horizontal and vertical lines that were gathered as two separate data sets. Due to these inconsistencies, the exact distortion cannot be quantified, but is easily visible in the mapping, along with anticlastic bending along the side edges. The mapping indicates an area of raised height in the crystal surface corresponding to a small bump on the edge of the bending frame window. This area matches exactly with the hole in the expanded beam and is believed to be the cause of the missing intensity.

## 5. Bent-crystal rocking-curve measurements (Bragg–Bragg mode)   

**Figure 6:**
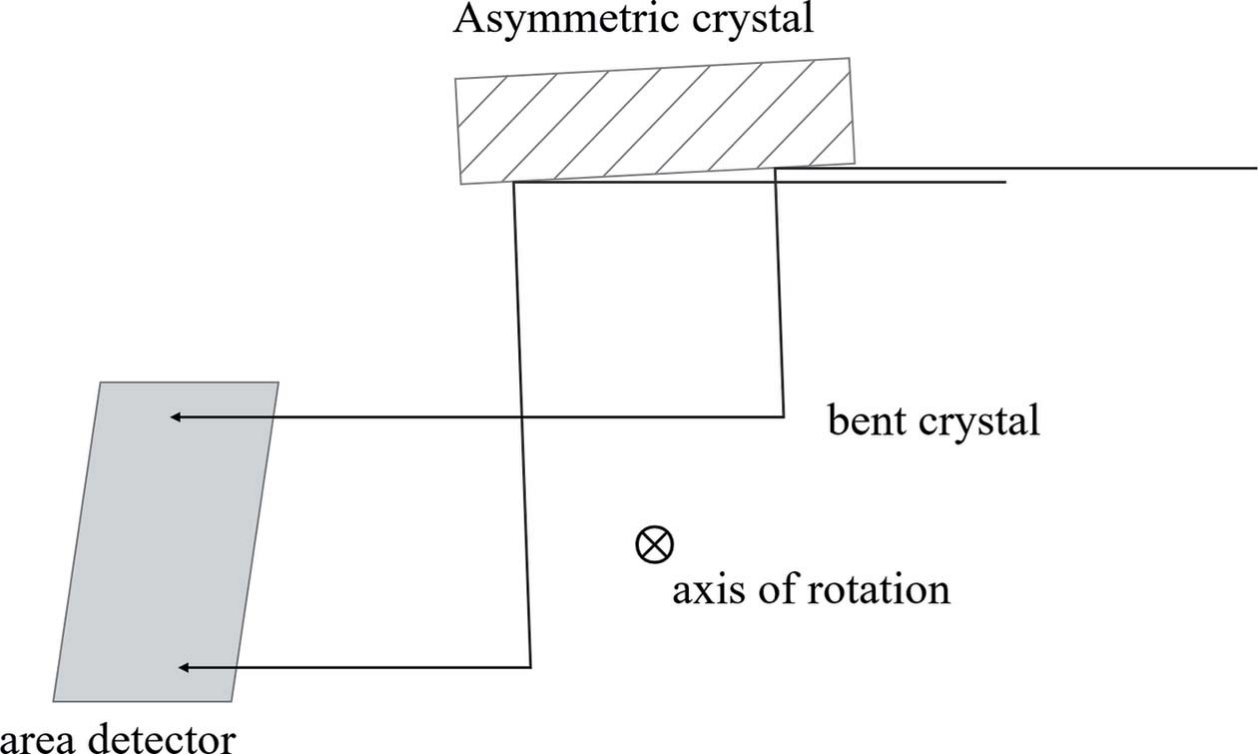
Variation of Berg–Barrett topography with bent crystal.

**Figure 7:**
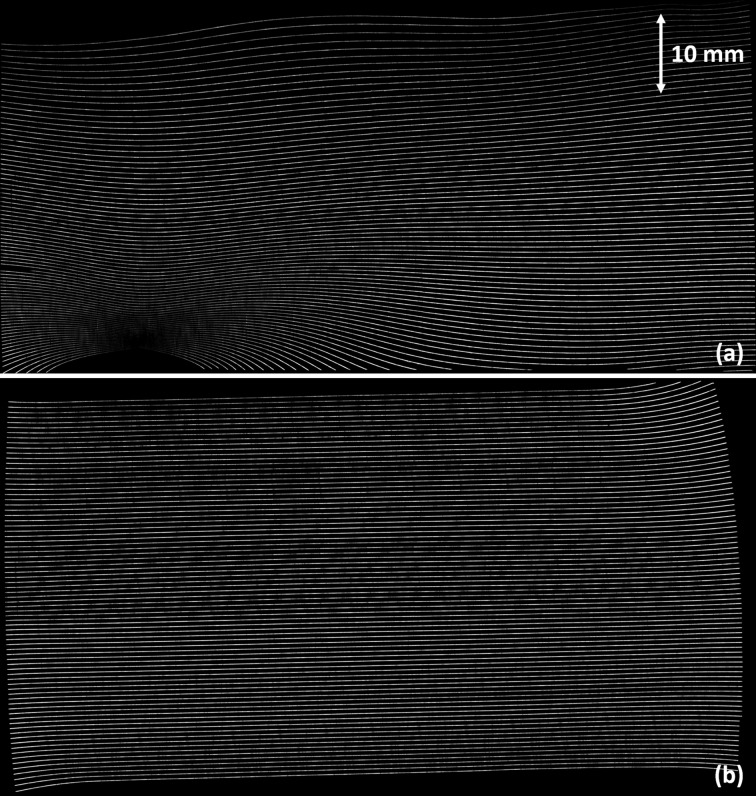
‘Zebra stripe’ images. (*a*) 5 m bend radius; note severe distortion in the lower-left corner and oscillating distortion along the top edge. (*b*) 0.5 m bend radius; anticlastic bending evident along sides and corners.

The first set of diffraction measurements was performed at the Advanced Photon Source Optics and Detector Testing beamline (Macrander *et al.*, 2016 ▸). An 8 keV monochromatic beam produced by the beamline’s double-crystal monochromator was conditioned by a flat Si(3,3,3) crystal that has an asymmetry angle of 46.6°. Using this expanded beam to completely flood the surface of the bent crystals in a variation of Berg–Barrett topography (Turner *et al.*, 1968 ▸), the 5 m and 0.5 m bend radius Si(5,1,1) crystals were rocked in 0.01° and 0.1° increments, respectively (Fig. 6 ▸). As the bent crystal is rotated in the diffraction plane, the monochromatic beam exiting the first crystal finds the matching Bragg planes at corresponding locations in the bent crystal, producing a map of the local curvature. After diffraction from the bent crystal, the X-ray beam was imaged using a Princeton Instruments PIXIS X-ray detector (13 mm × 13 mm field of view with 13 µm pixel size). These images were stitched together to form a ‘zebra stripe’ image for each crystal (Fig. 7 ▸). Each line represents an ‘isoangle’ line in the diffraction plane, *i.e.* the region of the crystal surface corresponding to a given diffraction angle for a fixed crystal orientation.

The Bragg–Bragg technique with a highly asymmetric conditioning crystal was chosen primarily for the low dispersion of the beam. The main disadvantage of this technique is that the expander setup uses the crystals in Laue–Laue diffraction mode, so there were concerns that the results may not be transferable. The technique also measures the convex side of the crystal whereas we expected most of the distortion to be introduced by contact between the bending frame and the concave side of the wafer. However, the excellent match between the regions of reduced intensity in the double-diffracted beam and the distortion in the crystal indicates that these concerns were unwarranted.

It was immediately clear that the region of missing intensity in the double-diffracted beam exiting the beam expander exactly corresponds to the region of severe distortion in the second crystal (5 m bend radius). The bending of the lines towards a common point indicates a convex deflection in the surface of the crystal, likely corresponding to a small bump in the mounting frame. The distortion along the top of this crystal also corresponds to a region along the top of the imaging beam that is similarly missing intensity. The first crystal (0.5 m bend radius) is relatively well bent. The effects of anticlastic bending are visible along the outer edge of the beam; however, the central region where the beam passes through is nearly perfectly bent, indicating that the regions of missing intensity in the expanded beam are nearly entirely caused by the second crystal.

**Figure 8:**
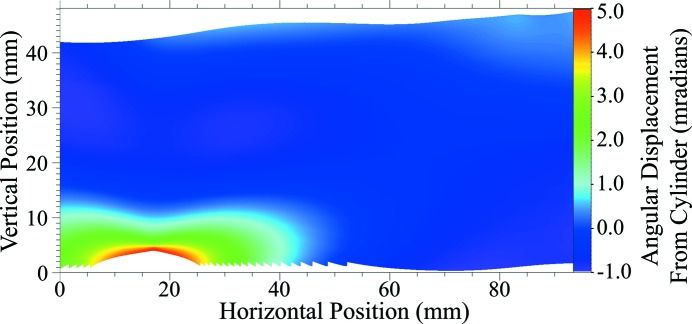
Angular distortion mapping of a 5 m bend radius crystal (colour bar scale in radians; *x* and *y* axes in mm).

The 5 m data were further analysed to quantify the angular deviation of the lattice planes in the diffraction plane from the expected diffraction angle at each position on the crystal surface. Using the known angular spacing of the lines (0.01°) and the known curvature of the crystal, we used bilinear interpolation to map the lines to a three-dimensional surface where the *z*-dimension represents relative diffraction angle as a function of (*x*, *y*) position in the crystal surface. A perfect cylinder was used to predict the theoretical diffraction angles. We measured the point-by-point deviation of our mapping from the prediction and mapped these to a coloured grid (Fig. 8 ▸). The bump reaches a maximum deviation of 4.4 mrad [much larger than the bandwidth of the first crystal as shown in Figs. 2(*b*) and 2(*c*) ▸], compared with an average deviation of 0.34 mrad in the areas of higher intensity. The large deviation of diffraction angle in the bump, corresponding to the red/yellow/green region on the mapping, correlates closely with the loss of intensity in the final beam, and areas of lesser distortion correlate with areas of lower intensity, as expected. When compared with Fig. 5 ▸, it is noted that the area of missing intensity is apparent in both images. However, as these images were produced using very different methods, there are some inconsistencies between the data sets. Specifically, the effects of anticlastic bending seem to be reduced in the diffraction mapping. This is to be expected, since anticlastic bending occurs perpendicular to the diffraction plane and hence has little effect on these measurements.

## 6. Bent-crystal rocking curves (Laue–Laue mode)   

**Figure 9:**
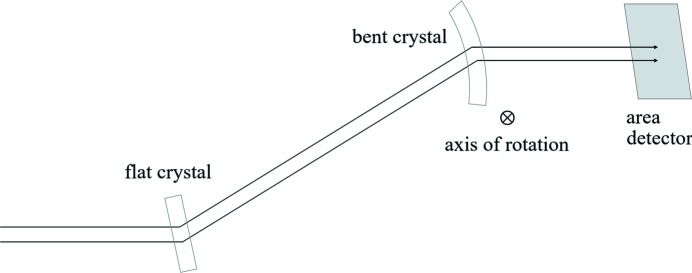
Variation of Lang topography with a bent crystal.

The second set of diffraction measurements was performed at the Canadian Light Source BMIT beamline. To match the diffraction conditions of the expander, these experiments were performed using Laue–Laue diffraction with a (3,1,1)-type reflection from a Si(5,1,1) wafer, just as in the expander experiments. In a variation of Lang projection topography (Lang, 1959 ▸), each bent silicon crystal wafer was rocked against a flat conditioning silicon crystal wafer (Fig. 9 ▸). As the bent crystal is rotated in the diffraction plane, the monochromatic beam exiting the first crystal finds the matching Bragg planes at different locations in the bent crystal, producing a map of the local curvature. The 5 m and 0.5 m bend radius wafers were rocked in 0.02° and 0.2° increments, respectively, and the axis of rotation was offset from the crystal surface so that local changes in curvature resulted in vertical displacements of the diffraction lines in the detector (Hamamatsu AA-60 beam monitor coupled to C9300-124 CCD camera with 8.75 µm pixel size).

**Figure 10:**
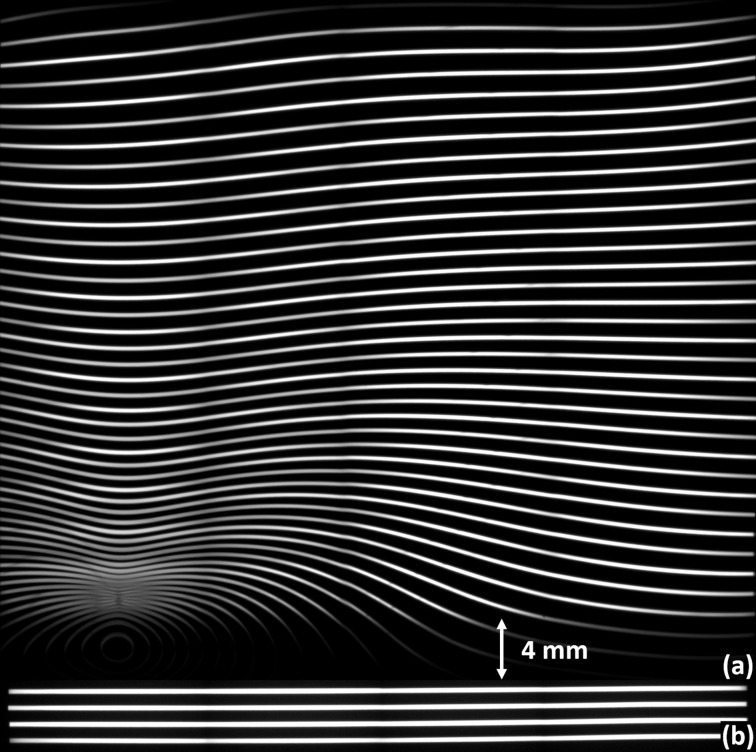
Laue–Laue bent crystal rocking curves. (*a*) 5 m bend radius; the areas of uniformly low intensity at the top and bottom of the 5 m image are caused by absorption in the aluminium frame. (*b*) 0.5 m bend radius; Laue-diffracted rays are nearly perfectly straight and parallel.

Reconstructed images are presented in Fig. 10 ▸. Here it is noted that for the 0.5 m bend radius the diffraction lines show only slight signs of anticlastic bending compared with the Bragg–Bragg measurements. This is primarily because the window in this frame limits the beam size, and within this region the anticlastic bending is virtually non-existent. This window also reduces the field of view of Fig. 10(*b*) ▸. Once again, it is observed that the beam produced by the first crystal is of excellent quality and that the areas of missing intensity in the final beam correspond exactly to the areas of distortion in the second crystal. This is reasonable given the large bend radius, as small distortions in the surface of the bending frame or crystal are proportionally much larger, magnifying their effects as compared with the 0.5 m bend.

## 7. Conclusion   

Several measurement techniques were used to characterize a bent Laue double-crystal beam-expanding monochromator used at the BMIT beamline at the Canadian Light Source. The physical measurements indicated that there was a region of severe distortion that appears to match up with the region of missing intensity in the final beam. Diffraction techniques in both Laue and Bragg geometry were used to create ‘zebra stripe’ images that clearly indicate a region of distortion in the second crystal. A novel analysis technique was developed to quantify the angular deviation of the lattice planes from the expected diffraction angle at each position on the crystal surface and thereby convert these stripe images into colour mappings representing the diffraction angle deviation. This technique could be used to analyse other bent crystals in synchrotron optical systems, for example to determine which crystal is distorted or whether these distortions are within some specified tolerance. In our case, we determined that the distortion of 4.4 mrad to be far outside our tolerance, as this caused a complete loss of intensity in the corresponding region of the beam.

## Figures and Tables

**Table 1:** Crystal parameters

Crystal type	Silicon (5,1,1)
Reflection type	(3,1,1)
Energy of experiments	33.2 keV
Bragg angle	6.55°
Crystal thickness	0.65 mm
First crystal bend radius	0.5 m
Second crystal bend radius	5 m

## References

[bb1] Lang, A. R. (1959). *Acta Cryst.* **12**, 249–250.

[bb2] Macrander, A., Erdmann, M., Kujala, N., Stoupin, S., Marathe, S., Shi, X., Wojcik, M., Nocher, D., Conley, R., Sullivan, J., Goetze, K., Maser, J. & Assoufid, L. (2016). *AIP Conf. Proc.* **1741**, 030030.

[bb3] Martinson, M., Samadi, N., Bassey, B., Gomez, A. & Chapman, D. (2015). *J. Synchrotron Rad.* **22**, 801–806. 10.1107/S1600577515004695 PMC4416688 25931100

[bb4] Martinson, M., Samadi, N., Belev, G., Bassey, B., Lewis, R., Aulakh, G. & Chapman, D. (2014). *J. Synchrotron Rad.* **21**, 479–483. 10.1107/S1600577514003014 PMC3998813 24763635

[bb9] Sanchez del Rio, M. & Dejus, R. J. (2011). *Proc. SPIE*, **8141**, 814115.

[bb8] Sanchez del Rio, M., Perez-Bocanegra, N., Shi, X., Honkimäki, V. & Zhang, L. (2015). *J. Appl. Cryst.* **48**, 477–491.

[bb5] Turner, A. P. L., Vreeland, T. & Pope, D. P. (1968). *Acta Cryst.* A**24**, 452–458.

[bb6] Wysokinski, T. W., Chapman, D., Adams, G., Renier, M., Suortti, P. & Thomlinson, W. (2007). *Nucl. Instrum. Methods Phys. Res. A*, **582**, 73–76.

[bb7] Zontone, F. & Comin, F. (1992). *Rev. Sci. Instrum.* **63**, 501–504.

